# Human Microbe-Disease Association Prediction Based on Adaptive Boosting

**DOI:** 10.3389/fmicb.2018.02440

**Published:** 2018-10-09

**Authors:** Li-Hong Peng, Jun Yin, Liqian Zhou, Ming-Xi Liu, Yan Zhao

**Affiliations:** ^1^School of Computer Science, Hunan University of Technology, Zhuzhou, China; ^2^School of Information and Control Engineering, China University of Mining and Technology, Xuzhou, China; ^3^Institutes of Science and Development, Chinese Academy of Sciences, Beijing, China

**Keywords:** microbe, disease, association prediction, adaptive boosting, decision tree

## Abstract

There are countless microbes in the human body, and they play various roles in the physiological process. There is growing evidence that microbes are closely associated with human diseases. Researching disease-related microbes helps us understand the mechanisms of diseases and provides new strategies for diseases diagnosis and treatment. Many computational models have been proposed to predict disease-related microbes, in this paper, we developed a model of Adaptive Boosting for Human Microbe-Disease Association prediction (ABHMDA) to reveal the associations between diseases and microbes by calculating the relation probability of disease-microbe pair using a strong classifier. Our model could be applied to new diseases without any known related microbes. In order to assess the prediction power of the model, global and local leave-one-out cross validation (LOOCV) were implemented. As shown in the results, the global and local LOOCV values reached 0.8869 and 0.7910, respectively. What’s more, 10, 10, and 8 out of the top 10 microbes predicted to be most likely to be associated with Asthma, Colorectal carcinoma and Type 1 diabetes were all verified by relevant literatures or database HMDAD, respectively. The above results verify the superior predictive performance of ABHMDA.

## Introduction

Microbes are ubiquitous in our lives. After deeper research, microbes could be simply divided into the following types: bacteria, fungi, viruses, archaea, protozoa, and so on (Sommer and Backhed, 2013). As we all know, there are a number of microbes living in the human tissues, such as gut (Grenham et al., 2011), skin (Fredricks, 2001) and lung (Cole, 1989). Cells are the basic unit of our body’s structure and function, and our body contains more than 40 trillion cells, but studies have shown that the number of microorganisms in humans is 10% more than the number of cells, which shows that the microbial community is relatively large in the human body (Sender et al., 2016). There are studies showing that microorganisms are involved in many biological processes in the human body, such as metabolic function, immune function, and so on (Gill et al., 2006). For example, in the intestinal tract of the adult, most of the intestinal microbes living in the gastrointestinal tract are able to not only synthesize necessary amino acids and vitamins, but also are conducive to the digestion and absorption of indigestible food (Huang Z.A. et al., 2017). So it is not surprising that there are links between microbes and human diseases (Consortium, 2012). Some researchers had found a close relationship between human type 2 diabetes and changes in the composition of the intestinal microbiota (Larsen et al., 2010). Gut microbes could induce colorectal cancer by generating butyrate that promoted the hyperproliferation of MSH2(-/-) colon epithelial cells (Belcheva et al., 2014). There was also evidence that toxins produced by microbes such as Streptococcus and Staphylococcus aureus had been shown to be a new class of allergens that could induce or even aggravate inflammatory skin diseases (Skov and Baadsgaard, 2000). Therefore, revealing disease-related microbes not only helps to further understand the pathogenesis of the disease but also provides new strategies for the diagnosis and treatment of the disease. Although some proven disease-microbe associations have been documented in the database HMDAD (Ma et al., 2017)^1^, such as Allergic asthma-Helicobacter pylori, Allergic sensitization-Clostridium difficile, and Asthma-Bacteroidetes, these are far from enough. Unfortunately, using biological experiments to reveal the relationship between disease and microbes is cumbersome and costly. Therefore, it is imperative to predict the potential disease-related microbes by constructing computational models.

According to the assumption that functionally similar microbes tend to be associated with similar diseases, by integrating two separate recommendation algorithms based on neighbor information and network topology, respectively, Huang Y.A. et al. (2017) developed a neighbor and graph based combined recommendation model for human microbe-disease association prediction (NGRHMDA) to predict potential disease-related microbes. As a combination of two independent recommendation models, the prediction accuracy of NGRHMDA was significantly improved compared to a single recommendation model. Unlike previous methods, NGRHMDA was an unsupervised learning method that did not require negative samples. Of course, there were some restrictions on NGRHMDA. Firstly, NGRHMDA could not be applied to predict microbes associated with new diseases without any known related microbes. Secondly, the optimal values of some parameters in the model were still not solved. Huang Z.A. et al. (2017) proposed a method of Path-Based Human Microbe-Disease Association prediction (PBHMDA) by integrating confirmed disease-microbe relations and the Gaussian interaction profile kernel similarity for diseases and microbes into a heterogeneous network. This model traversed all possible pathways between microbes and diseases through a novel depth-first search algorithm to predict the most likely disease-associated microbes. Both global and local leave-one-out cross validation (LOOCV) AUC values of PBHMDA were greater than 0.9, which showed that the prediction accuracy of PBHMDA was quite impressive. Regrettably, this model still had some shortcomings. Firstly, both the disease–disease similarities and microbe–microbe similarities were obtained from the Gaussian kernel for interaction profiles of microbes and diseases that were calculated based on the known disease-microbe associations, which might be biased for diseases with more known related microbes. Secondly, PBHMDA was also not suitable for new diseases. What’s more, based on the known human microbe-disease association network obtained from the HMDAD database, Wang et al. (2017) proposed a novel computational model of Laplacian Regularized Least Squares for Human Microbe-Disease Association (LRLSHMDA) to reveal potential disease-related microbes (Wang et al., 2017). LRLSHMDA applied a semi-supervised learning framework due to the lack of pairs of disease-microbes that had proven to be unrelated. In this model, the microbe similarity network and the disease similarity network were constructed based on the Gaussian interaction profile kernel similarity calculated by known microbe-disease association, and then by constructing and optimizing the cost functions in microbe space and disease space to integrated the optimal classifier functions to calculate the relation probabilities of microbe-disease pairs. Although the reliable prediction performance of LRLSHMDA had been verified, the model still had some shortcomings that needed further improvement. Firstly, the number of proven-microbe associations was too small, and sparse known association network might affect the prediction performance of the model. Secondly, LRLSHMDA could not be suitable for new microbes without any known related diseases.

In addition, Ma et al. (2017) built a microbe-disease association network based on published literature, and constructed a disease–disease network (Human Microbe Disease Network, HMDN) based on disease-associated microbes where the weight of the link between diseases was the similarity of microbes associated with the corresponding disease, and then by integrating data of disease genes, symptoms, chemical fragments, and drugs to investigate the overlaps between microbes and genes. Chen et al. (2017a) built a microbe-human disease association network and proposed a novel computational model of KATZ measure for Human Microbe-Disease Association prediction (KATZHMDA) based on this hypothesis that functionally similar microbes tend to have similar interactions and non-interactive patterns with non-infectious diseases and vice versa. By merging known disease-microbe association networks, disease similarity networks and microbe similarity networks into a heterogeneous network, KATZHMDA integrated walks with different lengths in the network to calculate the relation probability between microbe and disease. As a global computation method, KATZHMDA was capable of simultaneously revealing microbes associated with all diseases in a large-scale network. However, KATZHMDA still had many problems need to be solved in the future. For example, the problem of the optimal value of the parameter k had not been solved yet, and the prediction accuracy of KATZHMDA needed to be improved.

The above methods had various shortcomings. For instance, some models were not suitable for new diseases, and the optimal values of the parameters in some models were not well solved. For the sake of revealing the association between microbe-diseases better, in this paper, we proposed a model of Adaptive Boosting for Human Microbe-Disease Association prediction (ABHMDA) to uncover the associations between diseases and microbes by calculating the relation probability of disease-microbe pair using a strong classifier. Compared with the above methods, our model had the advantage of predicting microbes associated with new diseases. Since the number of negative samples was much larger than that of positive samples, we introduced k-means clusters to sample negative samples to balance the samples for training. What’s more, the strong classifier was composed of multiple weak classifiers according to the corresponding weights, and the higher the prediction accuracy of weak classifier, the greater the weight of it. We applied global and local LOOCV to evaluate the prediction performance of ABHMDA. As the results shown, the global and local LOOCV values reached 0.8869 and 0.7910, respectively, which indicated that the model’s prediction power was reliable. Besides, we used ABHMDA to conduct case studies on three diseases. 10, 10, and 8 out of the top 10 microbes predicted to be most likely to be associated with Asthma, Colorectal carcinoma and Type 1 diabetes were all verified by relevant literatures or database HMDAD, respectively.

## Materials and Methods

### Human Microbe-Disease Associations

We could obtain 450 known associations between 292 microbes and 38 diseases from Human Microbe-Disease Association Database (HMDAD) (Ma et al., 2017). For the reason that there were several grades of microbe classification, and when using 16s RNA sequences to study microbes, only the information in the level of genus would be acquired, we revealed the microbes which were likely to be related with human diseases in genus level. Besides, we defined the adjacency matrix A, if there was known association between disease *d*(*i*) and microbes *m*(*j*), the value of the element *A*(*d*(*i*), *m*(*j*)) matrix A was 1. We applied the variable nd, nm to denote the number of diseases and microbes studied, respectively.

### Gaussian Interaction Profile Kernel Similarity

Inspired by this article (Laarhoven et al., 2011), Considering the assumption that if two similar diseases were associated with two microbes, respectively, the two microbes were likely to be similar, and there were similar interaction and non-interaction pattern between diseases and microbes, Gaussian interaction profile kernel similarity for disease KD was constructed to indicated the similarities between diseases based on the known associations of disease-microbe pairs. Firstly, binary vector *IP*(*d*(*i*)) was defined to represented the interaction profiles of diseases *d*(*i*) by observing whether there was a known association between disease *d*(*i*) and each microbe (i.e., the ith row of the adjacency matrix A). Then, the Gaussian interaction profile kernel similarity between disease *d*(*i*) and *d*(*i*) could be calculated as follow:

(1) $$KD(d(i)\text{, }d(j))=\exp(-\gamma_{\text{d}}||IP(d(i))-IP(d(i))|{|}^{2})$$

Here, parameter γ_d_ was introduced to regulated the kernel bandwidth and got by normalizing another parameter γ′_d_ by the average number of related microbes of all the diseases. γ_d_ was calculated as follow:

(2) $$\gamma_{\text{d}}=\frac{\gamma_{\text{d}}^{\prime }}{\frac{\sum_{1}nd||IP(d(i))|{|}^{2}}{nd}}$$

where the value of γ′_d_ was 1.

The definition of Gaussian interaction profile kernel similarity for microbe KM was similar to KD

### Integrating Symptom-Based Disease Similarity

From the above we could see that Gaussian interaction profile kernel similarity was only based on the adjacency matrix A. If we wanted to effectively and scientifically predict potential disease-associated microbes, it was necessary to introduce other datasets in combination with the Gaussian interaction profile kernel similarity. Based on the disease and corresponding symptom recorded in PubMed bibliography. Zhou et al. (2014) calculated similarity between diseases and constructed the symptom-based human disease network (HSDN). Here, we integrated the Gaussian interaction profile kernel similarity for disease KD and the symptom-based disease similarity SDM to obtained the Integrating symptom-based disease similarity SD, and the calculation of SD was defined as follow:

(3) $$SD=\frac{KD+SDM}{2}$$

### ABHMDA

**FIGURE 1:**
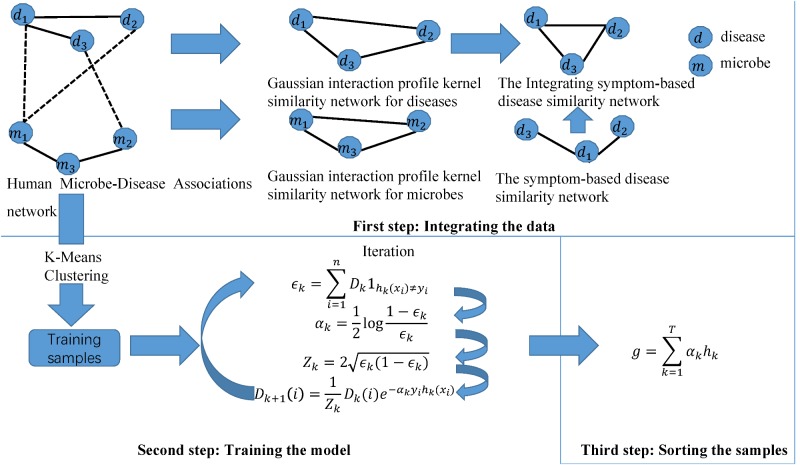
The flowchart of ABHMDA includes three steps: preparing the data; training the model; and scoring and ranking the disease-microbe pairs.

Motivated by this paper (Rayhan et al., 2017), we constructed a novel calculation model of ABHMDA to predict disease-related microbes and the flow chart of the algorithm was shown in **Figure 1**. The core idea of ABHMDA was to train different classifiers (weak classifiers) for the same training samples, and then grouped these weak classifiers with different ratios to form a stronger classifier to score and sort samples. Here, we chose the decision tree as our weak classifier. The specific steps were mainly divided into three steps: integrating the data, training the model, and scoring the samples. In the first step, we integrated the Gaussian interaction profile kernel similarity for microbe KM and the Integrating symptom-based disease similarity SD. In the second step, we firstly referred to the sample with confirmed association as a positive sample, otherwise it was an unknown sample. On account of the unknown sample accounting for about 97% in all the samples, that was to say, there were far more unknown samples than positive ones, and it was unreasonable to directly train such unbalanced datasets. Here, we introduced a novel method to balance the datasets. In this method, we applied the k-mean clustering to divide the unknown sample into k parts, and then randomly extract some samples from each part as negative samples, while positive samples kept unchanged. There were researchers studying the effect to random extraction when k took different values, and the results shown that the optimal value of parameter k was 23. In order to make the dataset used for training more balanced, the number of the unknown samples randomly selected ought to be approximately equal to the positive sample. In the end, the negative and positive samples together formed the training samples. Each training sample was weighted with an initial weight of $\frac{1}{n}$, where *n* was the total number of training samples. The main purpose of the training process was to calculate the proportion of each weak classifier in the final strong classifier and update the weight of each training sample according to whether it was classified correctly by the last classifier and the overall classification accuracy of the last classifier. After updating, the new training sample set with modified weight values was sent to the next weak classifier for training. Here, we built lists D_I_,*h*(*i*) and *Y* , all of which had n elements. The value of each element in D_i_ was the weight of the corresponding sample when the ith weak classifier trained the sample. The value of i was 0, 1, 2,,,,29. In other words, D_0_ was a list with all elements being $\frac{1}{n}$. The value of the element in label lists *h*(*i*) and Y was only 0 or 1, and the difference between them was that the value of *h*(*i*)_j_ depended on the prediction of the ith weak classifier, while the value of *Y*_j_ depended on whether the corresponding sample was a positive sample, if the corresponding sample was a positive sample, the value of *Y*_j_ was equal to 1, otherwise 0. The error function ∈_i_ was calculated as follow:

(4) $$\in_{\text{i}}\sum_{\text{j}=1}^{\text{n}}D_{\text{i}}1_{h(\text{i})j\neq Y_{\text{j}}}$$

It could be seen from the formula that the error function ∈_i_ was equal to the sum of the weights of the samples, whose label predicted by the weak classifier *h*(*i*)_j_ was different from the known label *Y*_j_. That was to say ∈_i_ was equal to the sum of the weights of all the samples that were predicted wrong. Then the proportion of the ith weak classifier in the strong classifier could be defined as follow:

(5) $$\alpha_{\text{i}}=\frac{\log\frac{1-\in_{\text{i}}}{\in_{\text{i}}}}{2}$$

It could be seen from equation (5) that the smaller the error function was, the larger the proportion of the weak classifier in the strong classifier would be. And the variate Z_i_ could be calculated as follow:

(6) $$Z_{i}=2{\left[\in_{\text{i}}(1-\in_{\text{i}})\right]}^{2}$$

The weight of the sample could be updated according to the following formula:

(7) $$D_{\text{i+1}}(j)=\frac{1}{Z_{\text{i}}}D_{\text{i}}\text{ (}j\text{)}e^{-\alpha_{\text{i}}Y_{\text{j}}h{(\text{i)}}_{\text{j}}}$$

Here *j* = 0, 1, 2...*n* - 1. After the weights of samples being updated, the samples with the new weights were sent to the next weak classifier to start the next training until all the weak classifiers completed the training (Theoretically, the more weak classifiers, the higher the prediction accuracy of strong classifier. But when the weak classifier reached a certain number, the prediction accuracy tended to be stable. And then as the number of weak classifiers increased, accuracy was not significantly improved, but the prediction process took longer. We compared the prediction results with 20, 30, and 40 weak classifiers, the accuracy of using 30 and 40 weak classifiers was basically the same, which was better than 20 weak classifiers. However, the prediction time of 40 weak classifiers was longer than using 30 classifiers. Comprehensive consideration of prediction time and accuracy, here, we chose to use 30 weak classifiers to form the final strong classifier.), then the training process was end. The next step was to score the sample, and the score of the jth sample was defined as follows:

(8) $$s(j)=\sum_{\text{i=0}}^{29}\alpha_{\text{i}}H{\left(i\right)}_{\text{j}}$$

Here, *HH*(*i*)_j_ was the score scored by the ith weak classifier for the jth sample. That was to say, the score of the sample was equal to the sum of the product of the sample’s goal scored by weak classifier and the corresponding weight (The corresponding data and code had been submitted to the website^2^).

## Results

### Performance Evaluation

In order to verify the prediction performance of ABHMDA, we implemented global and local LOOCV for our model based on the database HMDAD (Ma et al., 2017) which recorded 450 known associations between 39 diseases and 292 miRNAs. Specifically, each of the 450 samples (positive samples) with known association was left out in turn as a test sample while the remaining 449 were used for model training, while all of the samples without known associations were considered as candidate samples (unknown samples). In global LOOCV, we sorted the test sample with all candidate samples based on the score marked by calculation model, while the test sample was ranked with the candidate samples that contained the same disease as the test sample in local LOOCV. We evaluated the prediction performance of models based on the AUC value of the LOOCV. To be specific, only the test sample ranked above a certain threshold, could it be considered as a correct prediction, and then we set the true positive rate (TPR, sensitivity) as the horizontal axis and the false positive rate (FPR, 1-specificity) as the vertical axis. Therefore, we could plot the Receiver operating characteristics (ROC) curve, which was composed of points corresponding to different thresholds, then we could obtain the Area under the ROC curve (AUC). A model with an AUC value equal to 0.5 was equivalent to a random prediction. When the AUC took the maximum value of 1, the model had excellent prediction performance. In other words, when the value of AUC was greater than 0.5 and less than 1, the larger the value was, the better the prediction performance of the model would be.

**FIGURE 2:**
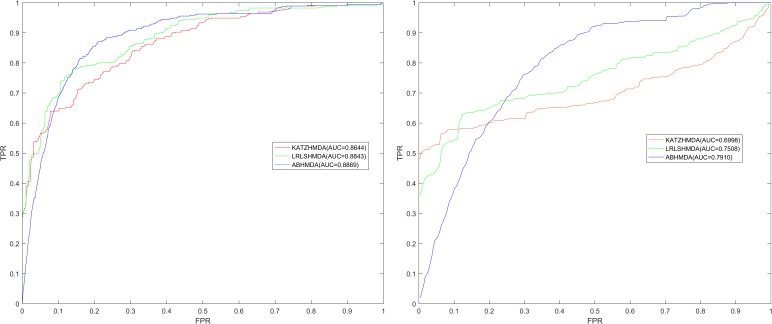
Comparison of prediction performance of ABHMDA with two other computational models (KATZHMDA, LRLSHMDA) in terms of ROC curves and AUCs values based on global and local LOOCV. As shown in the results, the global and local LOOCV values of ABHMDA were 0.8869 and 0.7910, respectively, which were significantly larger than that of KATZHMDA (0.8644, 0.6998) and LRLSHMDA (0.8843, 0.7508).

As shown in **Figure 2**, the global LOOCV value of ABHMDA was 0.8869, which was significantly larger than that of KATZHMDA (0.8644) and LRLSHMDA (0.8843). What was more, the local LOOCV value of our model reached 0.7910, which was also obviously better than KATZHMDA (0.6998) and LRLSHMDA (0.7508). These results confirmed the superior prediction performance of ABHMDA

### Case Study

In order to further assess the prediction ability of ABHMDA, we implemented two case studies on some important diseases of human. In the first kind, there were 10938 unknown samples about 39 diseases and 292 miRNAs in HMDAD. We sorted and ranked all unknown samples corresponding to the same disease and verified whether the association between the top 10 microbes and the disease studied was verified by the relevant literature. In the second kind, we converted all 1 in the adjacency matrix A to 0 and sorted all the samples (positive and unknown samples) corresponding to the same disease and then verified the association between disease and the 10 microbes most likely associated with it predicted by the model in the database HMDAD. In other words, the purpose of the second case study was to verify our model’s power to predict microbes associated with new diseases without any known related microbes. Here, we implemented the first case study on asthma, Colorectal carcinoma, and the second case on Type 1 diabetes.

As an inflammatory disease on the airway, it was very difficult to completely cure asthma under current medical conditions (Preston et al., 2007). According to statistics, there were about 300 million asthma patients worldwide, and in recent years its morbidity and mortality had also increased rapidly, especially in developing countries (Sagar et al., 2014). Therefore, a deeper study of asthma was imperative, and studies had shown that there was a close relationship between the microbes in the respiratory tract and the development and progression of asthma (Marri et al., 2013). For example, studies had shown that Firmicutes was reduced in asthmatic patients compared with normal humans (Wu et al., 2018). In contrast, Proteobacteria accounted for a larger proportion of microorganisms in asthma patients than normal people (Marri et al., 2013). What’s more, there was evidence that when the hypopharyngeal area of Neonates was infected with Streptococcus pneumoniae, the risk of developing asthma was increased compared to uninfected (Bisgaard et al., 2007). We implemented the first case study of asthma and the 10 microbes predicted to be most relevant to asthma were all verified by literatures. For instance, the experimental results showed that the abundance of Lachnospiraceae (First in the prediction list) in asthma patients was 1.9 times that of normal people (Jung et al., 2016). The researchers found that the relative abundance of Veillonella (Second in prediction list) in infants at risk of asthma was significantly lower than in normal people, and inoculation of sterile mice with Veillonella could improve its airway inflammation, which provided new ideas for the treatment of asthma (Arrieta et al., 2015). Moreover, there was evidence that if there was Clostridium coccoides (Third in prediction list) in a 3 week old baby’s stool, he was at risk of developing asthma, so Clostridium coccoides may become an early diagnostic target for asthma (Vael et al., 2011; See **Table 1**).

**Table 1:** The 10 microbes predicted to be most likely to be associated with the Asthma.

microbe	Evidece
Lachnospiraceae	PMID: 27433177
Veillonella	PMID: 26424567
Clostridium coccoides	PMID: 21477358
Firmicutes	PMID: 23265859
Streptococcus	PMID: 17928596
Actinobacteria	PMID: 23265859
Lactobacillus	PMID: 20592920
Bacteroides uniformis	PMID: 27433177
Enterococcus	PMID: 22641478
Escherichia coli	PMID: 26277095

*The first column records the top 10 microbes most likely to be related Asthma, and the second column records the databases and experimental literatures in PubMed, which verify the associations between the corresponding microbe and Asthma.*

To facilitate further research and validation, we provided a ranking of the relevant probabilities for all pairs of disease-microbe pairs without confirmed association (See **Supplementary Table S1**).

Colorectal carcinoma (CRC) was a common gastrointestinal malignant tumor in China (Xue et al., 2014). As one of the top cancers with the highest morbidity and mortality worldwide, it was estimated that there were approximately one million new cases of CRC and 500000 deaths per year (Sun et al., 2013). What was more serious was that its incidence would continue to increase in the next few decades, and the survival rate in 5 years was less than 60% (Sun et al., 2011). Therefore, it was necessary to study the pathogenesis of CRC to explored new treatment methods, and studies had shown that microbes played an important role in the development and progression of cancer that were closely related to inflammation like CRC (Liang et al., 2014). For example, there were studies showing that the number of Lactobacillus hamster increased significantly during the formation of CRC (Liang et al., 2014). The researchers compared CRC cases with the normal control group and found that the relative abundance of phylum Bacteroidetes in the case group reached 16.2%, which was much higher than 9.9% of the normal group (Ahn et al., 2013). We applied ABHMDA to implement the first case study on CRC, and the 10 predicted microorganisms most likely to be associated with CRC were all verified by related literature in PubMed. There was evidence that the relative abundance of Veillonella (First in the prediction list) in CRC cancer tissues was 2.87% and only 0.68% in the intestinal lumen (Chen et al., 2012). Pyogenic liver abscess was identified as an early manifestation of adult CRC, and an 11-year follow-up study showed that pyogenic liver abscess patients with Klebsiella (Second in the prediction list) pneumoniae had a higher probability of having CRC than those without (Huang et al., 2012). What was more, there were studies showing that Enterobacteriaceae (Third in the prediction list) was very rich in CRC patients (Arthur et al., 2014). From the above results, it could be seen that the predicted performance of ABHMDA was very reliable (See **Table 2**).

**Table 2:** The 10 microbes predicted to be most likely to be associated with the Colorectal carcinoma.

microbe	Evidece
Veillonella	PMID: 22761885
Klebsiella	PMID: 22776247
Enterobacteriaceae	PMID: 25182170
Proteobacteria	PMID: 24603888
Lachnospiraceae	PMID: 21850056
Clostridium coccoides	PMID: 19807912
Streptococcus	PMID: 21247505
Actinobacteria	PMID: 24316595
Lactobacillus	PMID: 15828052
Bacteroides uniformis	PMID: 24828543

*The first column records the top 10 microbes most likely to be related Colorectal carcinoma, and the second column records the databases and experimental literatures in PubMed, which verify the associations between the corresponding microbe and Colorectal carcinoma.*

Type 1 diabetes was an autoimmune disease which resulted from the immune-mediated destruction of insulin-producing pancreatic β cells (Li et al., 2014). The incidence of Type 1 diabetes was increasing globally, but the proportion of patients suffering from genetic factors was decreasing, which suggested that the virus, nutrition, and overweight were very likely to have become the main cause of Type 1 diabetes (Islam et al., 2014). Studies had shown that the abnormality in the gut microbiota was closely related to the development of Type 1 diabetes (De Goffau et al., 2014). The number of Firmicutes and Actinomycetes were significantly reduced in children with Type 1 diabetes compared with normal people (Murri et al., 2013). We conducted the second case study on Type 1 diabetes to test the prediction power of ABHMDA to predict the potential microbe-related of new diseases, and the results showed that 7 of the top 10 potential disease-related microbes predicted were validated by the database HMDAD. The associations between Type 1 diabetes and microbe Veillonella (First in the prediction list) with Bacteroidaceae (Second in the prediction list) were confirmed by HANDAD. Some researchers had found that patients with Type 1 diabetes had increased colonization of Enterobacteriaceae (Third in the prediction list) in addition to Escherichia coli compared with normal people (Soyucen et al., 2014). The above results indicated that ABHMDA’s ability to predict microbes associated with new diseases was also reliable (See **Table 3**).

**Table 3:** The 10 microbes predicted to be most likely to be associated with the Type 1 diabetes.

microbe	Evidece
Veillonella	confirmed
Bacteroidaceae	confirmed
Enterobacteriaceae	PMID: 24475780
Coxiellaceae	unconfirmed
Prevotella	confirmed
Bacteroidetes	confirmed
Prevotella copri	unconfirmed
Lachnospiraceae	confirmed
Lactobacillus	confirmed
Clostridia	confirmed

*The first column records the top 10 microbes most likely to be related Type 1 diabetes, and the second column records the databases and experimental literatures in PubMed, which verify the associations between the corresponding microbe and Type 1 diabetes.*

## Discussion

As a kind of tiny creature that are invisible to the human eyes, the microbes are small in size and simple in structure, but they are closely related to human beings. There are thousands of microbes in the human body. They build complex functional institutions and play an extremely important role in many biological processes, although they can benefit people, they can also bring a lot of trouble to human beings, such as diseases. More and more research shows that many human diseases are closely related to microorganisms, especially gastrointestinal diseases. Revealing the relation between disease and microbes contributes to further understand the pathogenesis of the disease and the development of new drugs (Chen et al., 2016b, 2017a). However, due to limited technology, the cost of using experimental methods to reveal disease-related microbes is greater. Therefore, it is imperative to construct model for the prediction of potentially relevant microbes. In this paper, we proposed a novel model ABHMDA to reveal the association between disease and microbes. The global and local LOOCV value of ABHMDA was 0.8869 and 0.7910, respectively, which was significantly larger than that of KATZHMDA (0.8644, 0.6998) and LRLSHMDA (0.8843, 0.7508). This result confirmed the strong prediction power of ABHMDA.

Several factors that led to ABHMDA prediction performance were summarized as follows. Firstly, the datasets used by our model were relatively reliable. Secondly, we extracted the potential similarities for diseases and microbes through Gaussian interaction profile kernel similarity. Thirdly, we combined multiple weak classifiers into one strong classifier according to different weights to score the samples. The high-precision weak classifiers accounted for a high proportion and vice versa, which conduced to improve the accuracy of the strong classifier. Of course, ABHMDA also had some defects that needed to be resolved in future work. Firstly, although the prediction performance of ABHMDA had improved compared to previous methods, prediction capabilities were expected to improve further if more reliable similarities were considered. Many groups have developed several effective computational models for the association prediction (Chen and Yan, 2013; Chen et al., 2016a; Chen and Huang, 2017; You et al., 2017; Chen et al., 2018a,b,c). We would introduce these reliable techniques to this new research area. Secondly, ABHMDA might cause bias to microbes with more associated diseases. Finally, the model did not consider the microbe–microbe similarity based on sequence similarity, which was also where we needed to improve in our future work (Chen et al., 2017b,c; Hu et al., 2018; Zhao et al., 2018).

## Author Contributions

L-HP and JY implemented the experiments, analyzed the result, and wrote the paper. LZ and M-XL analyzed the result and revised the paper. YZ conceived the project, developed the prediction method, designed the experiments, analyzed the result, and revised the paper. All authors read and approved the final manuscript.

## Conflict of Interest Statement

The authors declare that the research was conducted in the absence of any commercial or financial relationships that could be construed as a potential conflict of interest.

## Footnotes

1 http://www.cuilab.cn/hmdad

2 https://github.com/githubcode007/ABHMDA
