# Supplementary material for: Human Microbe-Disease Association Prediction Based on Adaptive Boosting
Source: Front Microbiol. 2018 Oct 9;9:2440. doi: 10.3389/fmicb.2018.02440 (PMC6189371; doi:10.3389/fmicb.2018.02440)
Supplement: Supplementary file 1 [file Table_1.DOCX]

Supplementary Material

Human microbe-disease association prediction based on adaptive boosting

**Li-Hong Peng^1,#^, Jun Yin^2,#^, Liqian Zhou^1^, Ming-Xi Liu^3,*^, Yan Zhao^2,*^**

^1^School of Computer Science, Hunan University of Technology, Zhuzhou, 412007, China

^2^School of Information and Control Engineering, China University of Mining and Technology, Xuzhou, 221116, China

^3^Institutes of Science and Development, Chinese Academy of Sciences, Beijing,100190, China

*** Correspondence:**Yan Zhao; Ming-Xi Liu

[ts17060090a3@cumt.edu.cn](mailto:ts17060090a3@cumt.edu.cn); liumingxi@casipm.ac.cn

**#Joint First Authors:**

Li-Hong Peng, Jun Yin

**Keywords: microbe; disease; association prediction; adaptive boosting; decision tree**

# Supplementary Table

**Supplementary Table 1.** We applied ABHMDA to prioritize all the candidate microbe-disease pairs based on all the known microbe-disease associations in HMDAD database as training samples. This prediction result is released for further experimental validation and research.
